# Global Prevalence of Eating Disorders in Nutrition and Dietetic University Students: A Systematic Scoping Review

**DOI:** 10.3390/nu15102317

**Published:** 2023-05-15

**Authors:** Sarah Budhiwianto, Christie J. Bennett, Claire A. Bristow, Janeane Dart

**Affiliations:** 1 Department of Nutrition, Dietetics and Food, School of Clinical Sciences, Monash University, Melbourne, VIC 3800, Australia; sarah.budhiwianto@monash.edu (S.B.); christie.bennett@monash.edu (C.J.B.); 2 Medical Education and Research Quality Unit (MERQ), School of Public Health and Preventive Medicine, Monash University, Melbourne, VIC 3800, Australia; claire.bristow@monash.edu

**Keywords:** eating disorders, disordered eating, body dissatisfaction, nutrition, dietetic education

## Abstract

Background: Nutrition and dietetics (ND) training encourages behaviors that can be considered risk factors for eating disorders or disordered eating. This paper aims to explore the prevalence of eating disorders (EDs) and predictors of eating disorders (/P-EDs) in ND students. Methods: A systematic scoping review of the literature was performed on PubMed, ERIC, PsychINFO, OVID Medline, and Scopus in October 2022. Results: A total of 2097 papers were retrieved from the search, of which 19 studies met the inclusion criteria. The resultant literature reported that 4–32% of ND students were at high risk of EDs (*n* = 6 studies), and 23–89% could be classified as having orthorexia nervosa (*n* = 7 studies). Further, 37–86% reported body image/fat dissatisfaction (*n* = 10 studies), and 100% of students reported weight dissatisfaction (*n* = 1 study). Conclusions: This paper highlights the prevalence of EDs and P-EDs across ND students. Further research is warranted to explore the cause, context, and impact on ND students’ wellbeing and professional identity and supporting diversity within the profession. Future studies should also consider curriculum approaches to address this occupational hazard.

## 1. Introduction

Nutritionists and dietitians are professionals who apply the science of food and nutrition to promote health, prevent and treat diseases, and help optimize the health of individuals, groups, and populations. Nutritionists and dietitians work across various settings, including private practice, community and public health, the food industry, research, sports, and primary care facilities such as hospitals [1]. To obtain a degree in nutrition and/or dietetics (N/D), a minimum of three undergraduate (UG) years of study are required, with many dietetic programs now offered as a two-year postgraduate (PG) program [2]. During their training, students are immersed in content exploring food composition; food science; body composition; and how to assess and modify dietary behaviors, attitudes, and patterns [3,4]. This focus on food and eating behaviors and patterns has the potential to be problematic for those students who may be vulnerable to disordered eating (DE) or eating disorders (ED).

Predictors of eating disorders (P-EDs) can include disordered eating and/or lack of intuitive eating, body dissatisfaction, and low levels of self-compassion [5]. Disordered eating exists across the spectrum between normal eating and diagnosed Eds [6,7]. Individuals with disordered eating behaviors experience symptoms and behaviors of EDs (e.g., risky eating behavior, binge eating, restrictive eating, compulsive eating, irregular eating patterns, and food addiction) but at a lower frequency and severity [6,7]. These symptoms can be an important key for early intervention and to stop the progression to an ED. EDs are serious, complex, and potentially life-threatening mental illnesses [8]. They are characterized by disturbances in behaviors, thoughts, and attitudes to food, eating, and body weight or shape. Eating disorders can have detrimental impacts on a person’s life and result in serious medical, psychiatric, and psychosocial consequences [8]. Various subtypes of EDs exist, as described in the *Diagnostic and Statistical Manual of Mental Disorders, Fifth Edition* (DSM-5) [9], including anorexia nervosa, bulimia nervosa, binge eating disorder, and other specified feeding and eating disorders (previously known as eating disorder not otherwise specified).

Nutrition and dietetic students are explicitly taught and expected to spend time understanding food and nutrition and the links to health and the body, and they consequently spend considerable time during their education thinking of food and dietary intake and ideas, thoughts, behaviors, and beliefs related to it [10,11]. Such behaviors can be seen as a preoccupation with food, which has been identified as a trait of disordered eating [12]. Furthermore, weight-centric ideologies are present in the ND pedagogy, such as weight biases or limited understandings of weight-neutral principles, existing in the professionals who teach the curricula [13,14,15]. It has been posited that some ND student cohorts have higher rates of EDs/P-EDs, but no research has been conducted from an international perspective to explore this issue [11]. It has been reported that up to 30% of ND students have entered the degree because they have experienced (including the experiences of family members and friends) having an ED or living in a larger body that is more likely to be pathologized [16]. While not yet formally recognized in the DSM-5, orthorexia nervosa is another ED that has become more pervasive in the ND profession [17]. It may be possible that the learning environments of ND curricula have the potential to exacerbate pre-existing or newly acquired ED/P-ED attitudes and behaviors [10,11]. However, this particular phenomenon has been deemed to be understudied, warranting further exploration [11]. Providing an environment where students could compassionately explore issues around food, body image, and stress management may help develop resiliency through professional practice [18]. Hence, it would be beneficial to explore how curriculum educational interventions can help address EDs/P-EDs in ND students.

The aim of this systematic scoping review was to explore the prevalence of EDs/P-ED in ND students.

## 2. Materials and Methods

### 2.1. Review Methodology

This systematic scoping review was undertaken according to the Preferred Reporting Items for Systematic Reviews and Meta-Analyses Extension for Scoping Reviews (PRISMA-ScR) checklist [19] and with reference to the JBI Manual for Evidence Synthesis [20].

### 2.2. Search Strategy

A systematic literature search was performed on 7th October 2022 on five databases, PubMed, ERIC, PsychINFO, OVID Medline, and Scopus, to identify eligible peer-reviewed journal articles. The PubMed search strategy was as follows: ((nutrition) OR (dietet *) AND (student *)) AND ((eating ADJ1 disorder *) OR (purg *) OR (laxative *) OR (binge *) OR (restrict *) OR (anorex *) OR (orthorex*) OR (bulimi *)). Search strategies were appropriately altered for specific database requirements such as word truncation and proximity operators. No search filters were used to collect all relevant papers. All results were uploaded to the referencing software Endnote X9.3.3 (Clarivate, Philadelphia, PA, USA) and then exported to Covidence Systematic Review Software (Veritas Health Innovation, Melbourne, Australia) where duplicates were removed before title and abstract screening.

### 2.3. Eligibility Criteria

The inclusion and exclusion criteria are listed in Table 1 as per the population, concept, and context (PCC) criteria suggested for systematic scoping reviews [20].

### 2.4. Study Selection

Both title and abstract screening and full text reviews were conducted regarding the eligibility criteria (Table 1) by two authors (SB and either CJB, CAB, or JD) to reduce selection bias and ensure consistency. Any conflicts were discussed between the researchers until a consensus was reached. Full texts were then retrieved and uploaded to Covidence. Publications that were in languages other than English were translated using two online translation programs, Google Translate (Google, Mountain View, CA, USA), and onlinedoctranslator.com (Ezoic Inc., Carlsbad, CA, USA).

### 2.5. Data Extraction

The characteristics of each study were extracted (e.g., country of origin, year, and study design), population characteristics (e.g., number of participants and demographic data), and conceptual factors (prevalence of EDs/P-EDs). Global cutoff points for each study were used to help determine the prevalence of the investigated outcome. If a study included the findings for a single outcome (e.g., body image dissatisfaction) with more than one category of intensity (i.e., slight, moderate, and severe risks), rates from each category were added together to obtain a total percentage rate for that one outcome. This total rate refers to the prevalence of students within the study who identified with that outcome. Additionally, only data that identified the prevalence of EDs and P-EDs in ND students were included in the results. Hence, if the eligible study also explored and included results from non-eligible cohorts (e.g., nutritionists) or included non-prevalence data (e.g., qualitative themes or mean quantitative scores without any clear cutoff points to indicate prevalence data), these findings were excluded from the data extraction process.

### 2.6. Synthesis of Results

Extracted data from the included studies are presented in Table 2. The characteristics of each paper are summarized alongside the instruments used and their specific findings related to the prevalence of EDs and P-EDs.

## 3. Results

### 3.1. Study Characteristics

**Figure 1:**
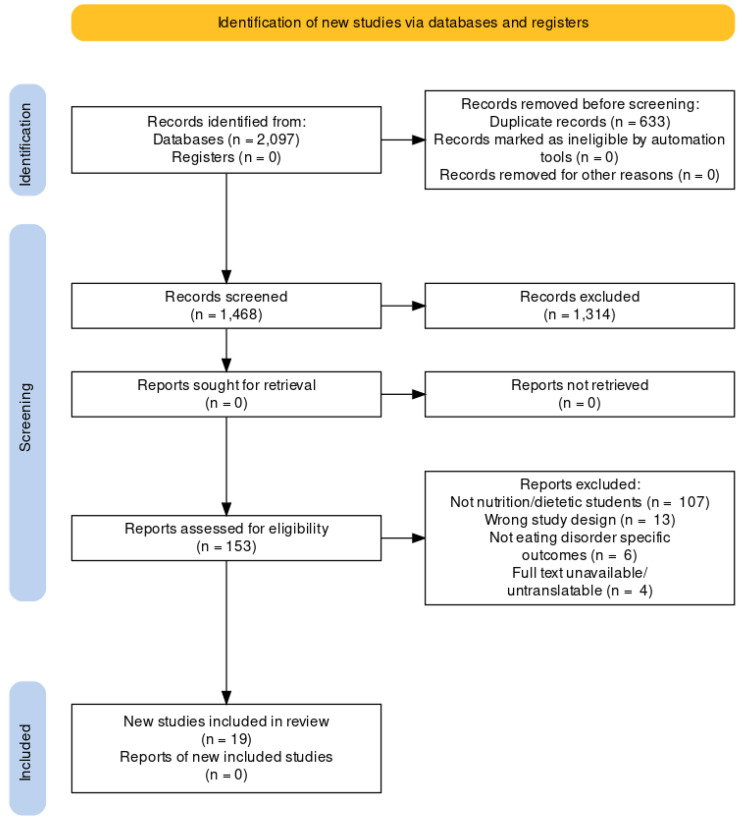
Preferred Reporting Items for Systematic Reviews and Meta-Analyses (PRISMA) flow diagram of included studies in the systematic scoping review.

A total of 2097 papers were retrieved from the search; of these, 19 studies were included in the systematic scoping review (Figure 1) [21,22,23,24,25,26,27,28,29,30,31,32,33,34,35,36,37,38,39]. Of the included studies, eleven were from South America [25,26,27,28,30,31,32,36,37,38,39], four were from Europe [23,24,33,34], three were from the Middle East [21,22,29], and one was from North America [35]. More than half of the included studies were exclusive female samples [22,23,24,26,27,28,32,33,37,38], whereas the remaining studies were mixed gender samples with female students representing 70–94% of the sample size [21,25,29,30,31,34,35,36,39]. Nearly all the included studies reported the mean age of the students, varying from 21 to 24 years [22,23,24,26,27,28,29,30,31,32,33,34,36,37,38,39]. Although most of the included studies lacked information on the level of study of the students included (UG or PG), eight studies mentioned they investigated students enrolled in UG degrees [25,26,28,29,32,34,36,38], with one study exploring both UG and PG students [21]. From the included studies that mentioned the student year levels they explored, one study specifically looked at first years [24], one looked at final years (not otherwise specified) [35], and seven studies explored first–third/fourth/fifth-year students [22,25,26,27,31,32,39]. Only one of the nineteen studies used a case–control design [33], whilst the remaining studies used a cross-sectional study design [21,22,23,24,25,26,27,28,29,30,31,32,34,35,36,37,38,39].

### 3.2. Prevalence of Eds/P-EDs in ND University Students

Table 2 summarizes the global prevalence of EDs/P-EDs in ND students. A case–control study found that nearly 18% of students met the lifetime criteria for any EDs [33]. Six cross-sectional studies found 4–32% of students were at high risk of EDs [22,27,30,31,37,38]. Further, a total of seven studies found that orthorexia nervosa was prevalent in 23–89% of ND students [21,22,29,32,34,36,39].

As for disordered eating, one cross-sectional study found that 5% of ND students had displayed food addiction [34]. Another cross-sectional study [35] found that 37% of students had risky eating behaviors, specifically, exercise, restrictive diets, binge eating, and feelings related to loss of control whilst eating.

Another P-ED behavior, body dissatisfaction, was found via 10 cross-sectional studies, which reported that body image dissatisfaction was prevalent in 37–86% of students [23,24,25,26,28,32,35,36,37,38]. Of these studies, two papers further identified that 71–75% of ND students displayed body fat dissatisfaction, whilst 100% of students had expressed some degree of body weight dissatisfaction [23,24].

### 3.3. Tools Used to Evaluate EDs/P-EDs

A total of 12 validated tools were used across the included studies to help explore the prevalence of ED/P-ED behaviors in ND students. To determine the prevalence of students at high risk of having EDs (anorexia nervosa, bulimia nervosa, binge eating disorder), 2 studies [22,27] used the original 40-question eating attitude test (EAT-40) [40], whilst 5 studies [29,30,31,37,38] used a shorter, 26-question eating attitude test (EAT-26) [41]. One study [33] used the Munich Composite International Diagnostics Interview (M-CIDI) [42] screening tool, which uses the definitions of EDs based on the DSM-5 [9] to assess the lifetime diagnosis of EDs (anorexia nervosa, bulimia nervosa, and eating disorders not otherwise specified). Three studies [21,32,36] used the ORTO-15 [43] tool to explore the prevalence of orthorexia nervosa, one of the ED subtypes. Similarly, three studies used the adapted version of the ORTO-15, the ORTO-11 [39,44], while one study [34] used the Bratman orthorexia test [45]. Disordered eating attitudes were explored via food addiction using the modified Yale food addiction scale (mYFAS) [46,47] in one study [34]. Another study [35] also assessed disordered eating through risky eating behaviors with the Brief Questionnaire of Risky Eating Behavior [48]. Finally, body dissatisfaction was explored using the Somatomorphic matrix [49] in two studies [32,35], silhouette scales [50] in another two studies [25,35], and the body shape questionnaire [51] in five studies [26,28,36,37,38]. See Appendix A for a detailed explanation of each tool and their cutoff points.

## 4. Discussion

The purpose of this review was to explore the prevalence of EDs/P-EDs in ND students. The resultant n = 19 studies show that there is a high prevalence of EDs/P-EDs in ND students. Further, the results suggest that ND students have a higher risk of EDs/P-EDs compared with other health professions. There is a dearth of literature investigating the impacts these outcomes have on professional identity and encouraging diversity within the profession. 

A range of ED/P-ED behaviors has been discovered in ND students, with orthorexia nervosa and body fat, body image, and body weight dissatisfaction being prevalent in more than 75% of the student cohorts. This dissatisfaction with self and, thus, attempts at control through the way these students perceive food may be explained by a previous literature exploration of the role that internalized fatphobia plays among dietitians [13]. Bessey et al. described how public perceptions of dietitians and dietetic pedagogy reflect that a dietitian ‘should be’ thin, healthy, and fit [13]. Furthermore, through the qualitative findings of da Silva et al., it was contextualized that ND students have expressed concerns that they have to adhere to the societal pressure of having a thin figure and live up to the perceived expectation of what a qualified nutritionist or dietitian ‘should’ eat or look like [30]. The influence of societal pressure that ND students may experience is further corroborated by Mahn and Lordly [11], who describe how the dietetic profession and society reinforce a relationship between the physical appearance of a dietitian and credibility. A 2021 study showed that when the word ‘dietitian’ was searched online, resultant images broadly depicted ‘thin, young, pretty, white and female’ individuals [52]. This representation of how members of the profession ‘look’ may result in ND students pressuring themselves into striving toward such ideological appearances in order to succeed in the profession [53,54]. This is a significant issue as students are exploring their professional identity, which may be limited if there is an incongruence between their bodies and the perceived standards [13,54]. This incongruence has been reported to impact perceptions of efficacy within the role of the dietitian and, potentially, job satisfaction [13]. There are also reported personality factors in ND students that may influence the risk of EDs/P-EDs, such as perfectionism, insecurity, and a lack of interoceptive awareness [27]. In the interest of supporting diversity and the longevity of practitioners within the profession, this highlights the need for pedagogical interventions to target subjects, such as regarding weight bias, perfectionism, and intrapersonal awareness.

In addition, Atkins and Gringas [54] postulated that, as ND students become increasingly familiar with the evidence-based practice of the profession, they may experience control discourse. Control discourse reflects the direct contrast between an individual’s eating patterns, which consist of a series of reasoned, discrete, and quantifiable choices (e.g., weighing, limiting, and avoiding foods) compared with eating that originates from hunger, appetite, emotions, and sociality [53]. Consequently, students may experience self-alienation and a transformation in their relationship with their bodies, food, and the interpersonal relationships they have with their families and friends [53,54]. This discourse may be a consequence of ND programs, which have a marked emphasis on science that sanctions a narrow range of perspectives of ‘healthy’ foods and body weights, apart from other dimensions of food and eating, which may intensify personality traits such as perfectionism [27]. Uncertainty tolerance is a concept whereby students are explicitly taught how to sit with the discomfort of not knowing. This may be a possible intervention target to reduce the control discourse in ND students [55].

It is important to note that the ranges for the prevalence rates of ED/P-ED outcomes were notably large. For instance, body image dissatisfaction (one of the P-ED outcomes) varied from 37 to 86% [23,24,25,26,28,32,35,36,37,38]. A possible explanation for these varying ranges may be the inconsistencies in the tools that were used. For example, body image dissatisfaction was investigated using three different tools, the Somatomorphic test [49], silhouette scales [50], and body shape questionnaires [51], which all have different methods of exploring the same outcome. Similarly, orthorexia nervosa rates ranged from 23 to 89% (a 66% variation) among the various ND students [21,22,29,32,34,36,39]. This may have occurred because various adaptations of the original ORTO-15 [43] have different cutoff points when investigating orthorexia nervosa risks. Therefore, for future research, it would be useful to utilize gold-standard methods such as the Eating Disorders Examination to help standardize results [56].

Several papers [57,58] also tried to investigate whether ED/P-ED tendencies among ND students differ compared with other university degrees. It was found that ND students were at an elevated risk because they had higher cognitive restraint scores, were more likely to be on a self-prescribed dietary regimen, had lower perceived ideal BMI, had reduced emotional eating [57], and had significantly higher orthorexia nervosa tendencies than other student cohorts [58]. However, body image dissatisfaction appears not to be an exclusive problem for ND students, as a study showed that scores did not significantly differ compared with physical education, advertising, and business administration students [59]. Regardless, this systematic scoping review highlights that body dissatisfaction (a part of P-EDs) and other ED behaviors are a concern for ND students. This is because ND students are later expected to enter the workforce as nutrition experts themselves, help solve nutritional problems, and support dietary and eating behavior changes that exist within communities and populations [1].

While ED/P-ED behaviors exist on a spectrum, it is important to consider that no degree of ED/P-ED is expected to be a part of an individual’s daily life [10]. Studies have shown that EDs and body dissatisfaction are risk factors for the development of mental health disturbances such as mood problems, social anxiety, depression, and suicidal ideation [60,61,62,63]. Hence, it is evident that EDs can have significant impacts on ND students’ physical and emotional wellbeing [64].

Thus, one potentially important way to help students better manage ED/P-ED behaviors would be through educational interventions embedded in the curricula that support positive mental health, body image, healthy relationships with food and eating, and wellbeing. 

No research to date has investigated this aspect. This absence of evidence and research highlights existing gaps in current ND curricula, which warrants action from ND university educators from around the world. Interestingly, previous research that examined the beliefs and approaches of EDs in ND education faculties around the world [65] found that 77% believed that EDs were an issue at their facility. However, only 15% had formal policies or procedures to address them [65]. Despite a desire for ND curricula reform [11,65], our systematic scoping review clearly indicates that there has not yet been any action or research documented within the current literature.

The previous literature has provided suggestions on how ND curricula can support students via educational interventions. Some studies have found that the increase in nutrition knowledge from the first to final years of ND degrees may influence students to adopt less restrictive tendencies, hence possibly impacting students’ ED/P-ED practices [66,67]. Furthermore, Agyopan et al. suggest that the design and delivery of the ND curriculum needs to address any nutritional misconceptions and flawed personal views that students may carry whilst they undertake the degree [22]. Incorporating a holistic approach toward nutrition within the curricula, such as Health at Every Size (HAES), may also be an effective way to impart positive attitudes and behaviors to ND students. Students who participated in a HAES general college course were found to have improved intuitive eating, body esteem, and anti-fat attitudes, as well as reductions in dieting behaviors when compared with students who completed a basic nutrition course [68].

Hence, this systematic scoping review reaffirms and supports the suggestions of Mahn and Lordly [11] on the need to create safe working and learning environments for ND students to openly discuss their ED/P-ED behaviors and food struggles, as well as space within curricula to explore these topics. Further research is still needed to better understand the etiology of ED/P-ED behaviors in the ND profession and how curriculum designers and educators can be provided with guidance in their subsequent attempts to address this challenge [10,11]. To help achieve this, it may be worthwhile to further explore the differences between ND students and other student cohorts.

This review has many strengths. Firstly, the review included the use of a systematic searching strategy, which ensures a rigorous and replicable review process. Secondly, this review provided a broad and robust search that did not include search limits to ensure international samples and prevalence could be captured. While this review has a strong methodology, there are some limitations. The present review has high heterogeneity between the included studies, whereby reporting measures and outcomes were often not consistent. Additionally, as a majority of the studies were cross-sectional in design, the inferences drawn from this review are somewhat limited by the quality of evidence of the included findings. However, this is a limitation of the resultant literature, not the search methodology, but it is important to keep in mind when interpreting the results. Further, online translation methods were also used to help translate non-English sources; hence, details and interpretation could have been lost in translation. However, it was important to capture an international sample. 

## 5. Conclusions

This review shows that 4–32% of ND students are at high risk for EDs; up to 89% meet the criteria for orthorexia, 18% meet the criteria for EDs within their lifetime, and up to 100% experience symptoms associated with disordered eating and P-EDs. Further research is still needed to explore the etiology of ED/P-ED behaviors within the profession and how this impacts professional identity and student wellbeing. Future studies should consider how curricula may be used to address this occupational hazard.

## Figures and Tables

**Table 1:** Inclusion–exclusion criteria for study selection.

	Inclusion	Exclusion
Population	- University undergraduate and/or postgraduate students of the ND curricula (e.g., nutrition science, human nutrition, health and nutrition, nutrition and dietetics, dietetics).	- Individuals who have graduated with an ND degree (i.e., new graduates, ND graduates, dietitians, participants in continuing professional development courses), as they are not part of the ND curricula anymore. - Students in general nutrition elective courses. They are excluded because the sample may be influenced by students from other university degree programs outside of the ND curricula who have also decided to undertake the course. - Students from other university degrees (e.g., nursing, occupational therapy, food sciences, etc.)
Concept	- Prevalence of EDs (e.g., anorexia nervosa, bulimia nervosa, binge eating disorder, other specific feeding or eating disorders, orthorexia nervosa). - Prevalence of P-ED behaviors (e.g., disordered eating, body dissatisfaction).	- Studies without any prevalence data or clear cutoff points for the instruments used. - Evaluation of eating competencies (separate from ED/P-ED behaviors) - Public health/widespread university actions that have not been specified to ND students.
Context	- Any ND university degree globally. - No language restrictions to maximize the reach of the study. - No year restriction (<7 October 2021) to maximize reach of study. - Studies that investigated prevalence data (e.g., cross-sectional studies).	- Narrative synthesis (i.e., systematic reviews), theses, and dissertations. - Comparison studies between students of ND vs. other courses.

Abbreviations: EDs: eating disorders, P-EDs: predictors of eating disorders, ND: nutrition and dietetics.

**Table 2:** Table of results for the global prevalence of eating disorder (ED) and predictors of eating disorder (P-ED) behaviors.

Author, Year of Publication	Country	Study Design	Degree Type and Year Level	N	Mean Age ± SD Years	Sex	Tool	Prevalence
Abdullah et al. [21]	Jordan	Cross-Sectional	Undergraduate + Postgraduate Student Year Level, N/A	385	18–22: 59% 23–30: 28% 31–40: 8% >40: 5% ^ † ^	70% F, 30% M ^ † ^:	ORTO-15	In total, 73% of undergraduates and 72% of postgraduates displayed orthorexia nervosa tendencies.
Agyopan et al. [22]	Turkey	Cross-Sectional	Degree Type, N/A, 1st–3rd-Year Nutrition Dietetic Students	136	21 ± 2	100% F	EAT-40 and Turkish Adaptation of ORTO-11	In total, 4% and 10% of students were at high and moderate risk of EDs, respectively; 71% of students displayed orthorexia nervosa tendencies.
Arroyo et al. [23]	Spain	Cross-Sectional	Degree Type, N/A Student Year Level, N/A	28	22 ± 2	100% F	Somatomorphic Matrix	In total, 68% of students had body image dissatisfaction; 75% of students were dissatisfied with their body fat, of which 29% were severely dissatisfied.
Arroyo et al. [24]	Spain	Cross-Sectional	Degree Type N/A	62	22 ± 2	100% F	Somatomorphic Matrix	A total of 69% of students had body image dissatisfaction, of which 37%, 15%, and 18% of students had slight, medium, and severe body image dissatisfaction, respectively.
			Last-Year Nutrition and Dietetic Students				Discrepancy between actual vs. ideal body weight	A total of 71% of students had body fat dissatisfaction, of which 37%, 18%, and 16% of students had slight, medium, and severe dissatisfaction with their body fat, respectively. In total, 100% of participants expressed some degree of body weight dissatisfaction, of which 68% wanted to weigh less, and 32% wanted to weigh more.
Alverenga et al. [25]	Brazil	Cross-Sectional	Undergraduate, 1st–3rd-Year Nutrition Students	629	<25 years: 55%, ≥25 years: 45% ^ ‡ ^	93% F, 7% M	Silhouette scales	In total, 86% of students experienced body dissatisfaction, of which 20% desired to have a larger body, whilst 66% desired a smaller body.
Bandeira et al. [26]	Brazil	Cross-Sectional	Undergraduate 1st–4th-Year Nutrition Students	300	24 ± 7	100% F	Portuguese Version of BSQ	A total of 47% of students had body image dissatisfaction, of which 29% were mild, 14% were moderate, and 4% were severe dissatisfaction.
Behar et al. [27]	Chile	Cross-Sectional	Degree Type, N/A 1st–4th-Year Nutrition Dietetic Students	123	21 ^ ‡ ^	100% F	Spanish Version of EAT-40	In total, 15% of students were at high risk of EDs.
Bosi et al. [28]	Brazil	Cross-Sectional	Undergraduate Student Year Level, N/A	193	21 ± 2	100% F	Portuguese Version of BSQ	A total of 40% of students had body image dissatisfaction, of which 22% were mild, 13% were moderate, and 6% were severe.
Caferoglu et al. [29]	Turkey	Cross-Sectional	Undergraduate Student Year Level, N/A	898	23 ± 5	91% F, 9% M	Turkish Version of EAT-26 and ORTO-11	In total, 32% of students were at high risk of EDs.In total, 64% of students displayed orthorexia nervosa tendencies.
da Silva Portonieri et al. [30]	Brazil	Cross-Sectional	Degree Type, N/A Student Year Level, N/A	809	24 ^ § ^	92% F, 8% M	Brazil Adaptation of EAT-26	In total, 15% of students were at high risk of EDs.
de Assis et al. [31]	Brazil	Cross-Sectional	Degree Type, N/A 1st–5th-Year Nutrition Students	201	22 ± 4	85% F, 15% M	Brazil Adaptation of EAT-26	In total, 28% of students were at high risk of EDs
de Souza et al. [32]	Brazil	Cross-Sectional	Undergraduate 1st–4th-Year Nutrition Students	150	23 ± 6	100% F	Portuguese version of ORTO-15	In total, 89% of students displayed orthorexia nervosa tendencies.
Frey et al. [33]	Germany	Case–Control	Degree Type, N/A Student Year Level, N/A	181	24 ± 3	100% F	DSM-5 AN, BN, EDNOS	Any EDs: 18%—anorexia nervosa: 7%, bulimia nervosa: 3%, eating disorder not otherwise specified: 7%.
Grammatikopoulou et al. [34]	Greece	Cross-Sectional	Undergraduate Student Year Level, N/A	176	22 ± 2	80% F, 20% M	BOT mYFAS	In total, 68% of students displayed orthorexia nervosa tendencies. 5% of students exhibited food addiction, a of disordered eating attitude.
Gutiérrez et al. [35]	Mexico	Cross-Sectional	N/A 1st-Year Nutrition Students	370	17–41 ^ ¶ ^	94% F, 7% M	Brief Questionnaire of Risky Eating Behavior and Silhouette Scales	In total, 37% of students were at risk of risky eating behavior, a disordered eating attitude, of which 25% and 12% of students were at moderate and high risk of risky eating behavior, respectively. The most frequent risky eating behaviors were exercise, restrictive diets, binge eating, and feelings of loss of control when eating. In total, 72% of students had body image dissatisfaction; 65% and 8% of students wanted to have slimmer or robust body silhouettes, respectively.
Penaforte et al. [36]	Brazil	Cross-Sectional	Undergraduate Student Year Level, N/A	141	22 ± 4	91% F, 9% M	Portuguese Version of ORTO-15 BSQ	In total, 87% of students displayed orthorexia nervosa tendencies. A total of 58% of students had some degree of body image dissatisfaction, ranging from mild to severe. A specific % breakdown of each dissatisfaction category was not stated in the paper.
Silva et al. [37]	Brazil	Cross-Sectional	Degree Type, N/A Student Year Level, N/A	175	22 ± 2	100% F	Portuguese Version of EAT-26 BSQ	In total, 22% were at high risk of EDs. A total of 37% of students had body image dissatisfaction, of which 23% were mild, 8% were moderate, and 6% were severe dissatisfaction.
Toral et al. [38]	Brazil	Cross-Sectional	Undergraduate Student Year Level, N/A	427	23 ± 5	100% F	Portuguese Version of EAT-26 and BSQ	In total, 10% of students were at high risk of EDs. A total of 54% of students had body image dissatisfaction, of which 19% were mild, 8% were moderate, and 27% were severe dissatisfaction.
Villa et al. [39]	Chile	Cross-Sectional	Degree type N/A, 1–5th-Year Nutrition Dietetic Students	90	22 ± 3	88% F, 12% M	Spanish Version of ORTO-11 (with a ≤27 cut point)	In total, 23% of students were at risk of orthorexia nervosa.

^†^ Data were calculated with the rest of the participants, including graduates and nutritionists. ^‡^ Data presented as a percentage proportion of the total cohort. ^§^ Nil SD reported. ^¶^ Data presented as a range. Abbreviations: F: female, M: male, SD: standard deviation, N/A: information not available, EDs: eating disorders, EAT: eating attitude test, BSQ: body shape questionnaire, BOT: Bratman orthorexia test, mYFAS: modified Yale food addiction scale, DSM-5: *Diagnostic and Statistical Manual of Mental Disorders, Fifth Edition*, AN: anorexia nervosa, BN: bulimia nervosa, EDNOS: eating disorder not otherwise specified.

## Data Availability

Not applicable.

## Appendix A

**Table:**

Name	Details	Tests
Eating Attitude Test: EAT-40 [40] EAT-26 [41]	- Both the long and short versions of the eating attitude test (EAT-40 and EAT 26, respectively) have been used to identify an individual’s ED risk based on their behaviors, feelings, and attitudes associated with eating. - EAT-40, which is a 40-item objective questionnaire, was initially developed by Garner and Garfinkel in 1979 to evaluate symptoms of anorexia nervosa according to seven factors: pre-occupation with food, thin ideal body image, dieting, vomiting/laxative abuse, slow eating, clandestine eating, and social pressure to gain weight. A cutoff score of ≥30 is considered a high risk for EDs, and 21–30 is a moderate risk. - In 1982, Garner created and validated EAT-26, a shorter version of the test that can be used to screen EDs related to anorexia nervosa, bulimia nervosa, and binge eating disorder. This scale includes 26 self-report questions assessing general eating behaviors and an additional 5 questions related to risky eating behaviors related to 3 subscales: dieting, bulimia, and food pre-occupation and oral control. A total score of 0–78 indicates an overall risk score, with a higher score indicating a greater risk of EDs. A cutoff >20 is considered a high risk of an ED.	EDs—Anorexia Nervosa, Bulimia Nervosa, Binge Eating Disorders
Munich Composite International Diagnostic Interview (M-CIDI) [42]	M-CIDI is a screening tool that can be used by trained interviewers to help assess psychiatric disorders based on the definitions and criteria of the International Classification of Disease (ICD-10) and the Diagnostic and Statistical Manual of Mental Disorders (DSM-IV).	EDs—Anorexia Nervosa, Bulimia Nervosa, Eating Disorder Not Otherwise Specified (Atypical Anorexia Nervosa/Bulimia Nervosa, Binge Eating Disorder)
ORTO-15 and ORTO-11 [43,44]	ORTO-15 and its adapted version ORTO-11 are, respectively, 15- and 11-item questionnaires that assess orthorexia tendencies, i.e., attitudes related to food selection, the extent to which concerns over food influence daily life, the perceived effects of eating healthy food, and dietary/eating habits. Each item has a score of 1–4, where 1 indicates orthorexia tendencies, and 4 indicates normal eating behavior. A total of 60 points can be obtained for ORTO-15 and 44 points for ORTO-11. A low score indicates orthorexia tendencies, with cutoff points for being symptomatic of ON for ORTO15 being ≤40 and ORTO-11 being ≤27.	EDs—Orthorexia Nervosa
Bratman Orthorexia Test (BOT) [45]	Ten dichotomous items with Yes or No answers. One point is given for every positive answer. Scores < 5 classify as healthy, scores of 5–9 classify as health fanatics, and scores of 10 classify as having orthorexia nervosa.	EDs—Orthorexia Nervosa
Modified Yale Food Addiction Scale (mYFAS) [46,47]	Nine-item questionnaire that assesses food addiction and its symptoms. Seven items assess one of the seven symptoms of substance dependence according to the DSM-IV criteria (control, attempts, time, activities, problems, tolerance, withdrawal, and impairment) and two domains evaluate the presence of a clinically significant impairment of distress. Food addiction can be ‘diagnosed’ when at least three symptoms and a criterion of clinically significant impairment or distress was met.	P-EDs—Food Addiction
Brief Questionnaire of Risky Eating Behavior [48]	Self-administered questionnaire consisting of 10 items that assess fear of gaining weight, binge eating, loss of control while eating, self-induced vomiting, fasting, dieting, excessive exercising, diet pill use, diuretics use, and laxative use in the past 3 months. Each item has 4 response options: 0 = never, 1 = sometimes, 2 = frequently (2 × week), and 3 = very frequent ( >2 × week). Scores are added together, with higher scores indicating increasing severity of symptoms. A score of >10 refers to being at high risk of risky eating behaviors.	P-EDs—Risky Eating Behavior
Silhouette Scales [50]	Participants are provided with nine sets of female and male body silhouettes with varying body dimensions and shapes ranging from a thin to a large silhouette. Participants are then asked to identify which silhouette they perceive themselves to be. Those who claim to have a silhouette greater or lesser than the silhouette which corresponds to the participant’s actual BMI are considered to have body image dissatisfaction.	P-EDs—Body Dissatisfaction (specifically image)
Somatomorphic Matrix [49]	A computer-based test. Two standard questions are asked: 1. Choose the image that best represents your own body (actual body image), and 2. choose the image that represents the body that you would ideally like to have (ideal body image). Participants are left to scroll through images until they have selected a ‘best-fit’ answer. The degree of dissatisfaction is measured with four categories based on the classification made by Casillas-Estrella et al. [69] and differences between the Fat-Free Mass Index (FFMI, 1.5 kg/m2) and body fat (4%) in images from the Somatomorphic matrix test. The participants are considered to be satisfied with their body images if the difference between their actual vs. ideal body image is zero. The other categories include 1 = slight dissatisfaction (difference of 1.5 kg/m2 in FFMI and 4% in body fat); 2 = moderate dissatisfaction (difference of 3.0 kg/m2 in FFMI and 8% in body fat), and 3 = severe dissatisfaction (difference of 4.5 kg/m2 in FFMI and 12% in body fat).	P-EDs—Body Dissatisfaction (specifically, image and fat)
Body Shape Questionnaire (BSQ) [51]	A 34-item self-report questionnaire that measures concerns over body shape and body image dissatisfaction in general. Responses to each item ranged from 1 = never to 6 = always. Cutoff points are as follows: <80 = absence of BID, 80–110 = mild dissatisfaction, 111–140 = moderate dissatisfaction, and >140 = severe dissatisfaction.	P-EDs—Body Image Dissatisfaction

Abbreviations: EDs: eating disorders, P-EDs: predictors of eating disorders, M-CIDI: Munich composite international diagnostic interview, EAT: eating attitude test, ICD-10: International Classification of Disease (ICD-10), DSM-IV: *Diagnostic and Statistical Manual of Mental Disorders, 4th Edition*, BOT: Bratman orthorexia test, mYFAS: modified Yale food addiction scale, BSQ: body shape questionnaire, BMI: body mass index, FFMI: fat-free mass index.
